# MicroRNA-186 targets IGF-1R and exerts tumor-suppressing functions in glioma

**DOI:** 10.3892/mmr.2022.12778

**Published:** 2022-06-20

**Authors:** Jian Jiang, Weijie Wang, Dazhao Fang, Xiaodong Jin, Lianshu Ding, Xiaoyang Sun

Mol Med Rep 16: 7821–7828, 2017; DOI: 10.3892/mmr.2017.7586

Following the publication of this paper, it was drawn to the Editors’ attention by a concerned reader that certain of the cell migration assay data shown in Fig. 2C were strikingly similar to data that had appeared in different form in another article by different authors. Owing to the fact that the contentious data in the above article had already been published elsewhere prior to its submission to *Molecular Medicine Reports*, the Editor has decided that this paper should be retracted from the Journal. After having been in contact with the authors, they agreed with the decision to retract the paper. The Editor apologizes to the readership for any inconvenience caused.

